# Patient-reported outcomes in patients with overactive bladder treated with mirabegron and tolterodine in a prospective, double-blind, randomized, two-period crossover, multicenter study (PREFER)

**DOI:** 10.1186/s12955-018-0892-0

**Published:** 2018-04-19

**Authors:** Sender Herschorn, David Staskin, Le Mai Tu, Jonathan Fialkov, Terry Walsh, Katherine Gooch, Carol R. Schermer

**Affiliations:** 1 Sunnybrook Health Sciences Centre, University of Toronto, 2075 Bayview Avenue, MG408, Toronto, Ontario M4N 3M5 Canada; 2 Tufts University School of Medicine, Boston, MA USA; 3 University of Sherbrooke, Quebec, Canada; 4 The Iowa Clinic, West Des Moines, IA USA; 5 Astellas Pharma US, Northbrook, IL USA

**Keywords:** Mirabegron, Tolterodine ER, Overactive bladder, OAB-questionnaire, Health-related quality of life, OAB-satisfaction, Patient Perception of Bladder Condition, Patient-reported outcomes

## Abstract

**Background:**

The PREFER study was an assessment of medication tolerability, treatment preference and symptom improvement during treatment with mirabegron (M) and tolterodine (T) extended release (ER) in patients with overactive bladder (OAB). In this analysis of PREFER, patient-reported outcomes (PROs) were assessed during treatment.

**Methods:**

PREFER was a two-period, 8-week crossover, double-blind, phase IV study (NCT02138747) of treatment-naïve adults with OAB ≥3 months randomized to 1 of 4 treatment sequences (M/T; T/M; M/M; T/T), separated by a 2-week washout. Tolterodine ER was dosed at 4 mg for 8 weeks and mirabegron was dosed at 25 mg for 4 weeks then increased to 50 mg for the next 4 weeks. At each visit, PROs related to treatment satisfaction, quality of life and symptom bother were assessed using the OAB Satisfaction (OAB-S; 3 independent scales/5 single-item overall assessments), OAB-q (total health-related QoL [HRQoL] and subscales [Sleep, Social, Coping, Concern] and Symptom Bother scale) and Patient Perception of Bladder Condition (PPBC) questionnaires. Responder rates were reported for OAB-q subscales based on a minimal important difference (MID; ≥ 10-point improvement) and OAB-S Medication Tolerability score ≥ 90.

**Results:**

In total, 358 randomized patients received ≥1 dose of double-blind study medication and completed ≥1 post-baseline value (OAB-S scale, OAB-q, PPBC): M/T (*n* = 154), T/M (*n* = 144), M/M (*n* = 30) or T/T (*n* = 30). At end of treatment (EoT), mirabegron and tolterodine ER were associated with similar mean improvements in 7 of the 8 OAB-S scores investigated, OAB-q scales and PPBC. A higher percentage of patients achieved clinically relevant improvements (MID) in OAB-q scales and OAB-S Medication Tolerability score during treatment with mirabegron than tolterodine ER.

**Conclusions:**

On average, patients with OAB experienced improvements in treatment satisfaction, HRQoL and symptom bother that were of a similar magnitude during treatment with mirabegron or tolterodine ER. However, during mirabegron treatment, patients were more likely to achieve clinically relevant improvements in tolerability and HRQoL (as measured by the MID for the OAB-q or an OAB-S Medication Tolerability score ≥ 90) than during tolterodine ER treatment.

**Trial registration:**

NCT02138747; registered May 13, 2014.

## Background

Overactive bladder (OAB) is a syndrome, comprising urinary urgency, usually accompanied by increased daytime frequency and nocturia, with or without urinary urgency incontinence, in the absence of urinary tract infection or other obvious pathology [1, 2]. The prevalence of OAB increases with age and is expected to affect 1 in 10 people by 2018 [3]. The chronic nature of OAB and severity of symptoms makes it problematic for many patients, often resulting in significant deterioration in quality of life (QoL), depression and social isolation [4]. Significant economic consequences are associated with OAB as a result of health resource costs and decreased work productivity [4].

Patients with OAB tend to seek treatment once their QoL is affected, and are more likely to persist with their medication if they perceive meaningful improvements in QoL [5]. This underlies the importance of evaluating the benefits of a treatment not only according to objective changes in bladder parameters (e.g. micturition frequency, incontinence episodes) but also via subjective outcomes related to QoL, perception of symptoms, and general well-being. Validated bladder health questionnaires include the OAB-questionnaire (OAB-q), Patient Perception of Bladder Condition (PPBC), and OAB-Treatment Satisfaction (OAB-S) questionnaire. The OAB-q is useful for assessing treatment effects on various aspects of QoL such as social interaction, coping, sleep, and the extent of bother associated with symptoms [6, 7], and the single-item PPBC evaluates patients’ perception of their current bladder problems [8]. However, neither the OAB-q or PPBC explores additional factors related to patient satisfaction with their medication. The OAB-S was developed to measure the ‘multidimensional concept’ of treatment satisfaction over a number of domains, including 5 independent scales (OAB Control Expectations, Impact on Daily Living with OAB, OAB Control, OAB Medication Tolerability, and Satisfaction with OAB Control) and 5 single-item overall assessments (Patient’s Fulfillment of OAB Medication Expectations, Interruption of Day-to-Day Life Due to OAB, Overall Satisfaction with OAB Medication, Willingness to Continue OAB Medication, and Improvement in Day-to-Day Life Due to OAB Medication) [9].

Communicating the clinically meaningful benefits of treatment to the patient can be simplified through the use of responder analyses [10]. These clinically meaningful changes are often expressed as specific differences or thresholds, known as a minimally important difference (MID). The MID is assessed at the individual level of analysis such that changes consistent in magnitude with the MID in individual PROs over time are interpreted as a treatment benefit in the population [10]. The MID is defined as “the smallest difference in score in the domain of interest that patients perceive as beneficial and which would mandate, in the absence of troublesome side effects and excessive costs, a change in patient management” [11]. This approach is used to categorize patients into two distinct groups based on those who attain a treatment benefit in the PRO (‘responder’) vs those who do not attain a treatment benefit (‘non-responder’). For PROs in which no MID has been established, responders to OAB treatment have been variably defined and have included positive response categories to specific questionnaire items [12].

Mirabegron (β3-adrenoceptor agonist) and tolterodine (antimuscarinic) belong to the two classes of oral pharmacotherapies used to treat OAB. Both drugs have similar efficacy in decreasing OAB symptoms of urinary urgency, frequency and incontinence. The different mechanism of action of mirabegron, however, is associated with a lower frequency of specific antimuscarinic side effects, such as blurred vision and dry mouth [13]. Dry mouth is the most frequent and bothersome side effect reported with antimuscarinics, [14] and one of the main reasons patients discontinue antimuscarinic treatment [15].

Potential differences in tolerability between antimuscarinics and mirabegron may confer clinically meaningful improvements in health-related QoL (HRQoL), treatment satisfaction, and persistence. Medication tolerability can be an issue in both treatment-experienced and treatment-naïve patients; however, the treatment-naïve patients have lower rates of persistence at 12 months, [16] which may be a consequence of higher treatment expectations or a lower tolerability threshold compared with treatment-experienced patients.

In clinical practice, the successful management of OAB demands greater focus on demonstrable benefits in PROs rather than relying solely on reductions in bladder symptoms. Each patient is different in terms of how he or she experiences symptoms and responds to treatment, according to his or her own priorities, expectations, and attitudes. Therefore, a comprehensive approach to evaluating efficacy and tolerability that considers the impact of symptoms on the individual’s experience and lifestyle may be predictive of long-term persistence [17].

A crossover study design is a methodology for comparing two or more therapies in the same patient and obviates the need to account for inter-patient variability. PREFER was a phase IV, crossover trial in patients with OAB in which a statistically significantly higher OAB-S Medication Tolerability score (the primary endpoint), implying better tolerability, was demonstrated with mirabegron vs tolterodine extended release (ER) 4 mg [18]. Mirabegron was also associated with significantly fewer anticholinergic side effects [18]. However, improved tolerability did not translate into preference for mirabegron (the secondary endpoint). Herein PROs and corresponding responder rates from the PREFER study are reported to further explore potential treatment differences and identify outcomes that may be predictive of treatment success.

## Methods

### Study design

PREFER (ClinicalTrials.gov NCT02138747) was a prospective, randomized, multicenter, double-blind, higher order (i.e. more periods/sequences than number of treatments being compared [19]), two-period crossover, phase IV study, conducted in 36 sites (28 sites in the United States and 8 sites in Canada) [18].

**Fig. 1:**
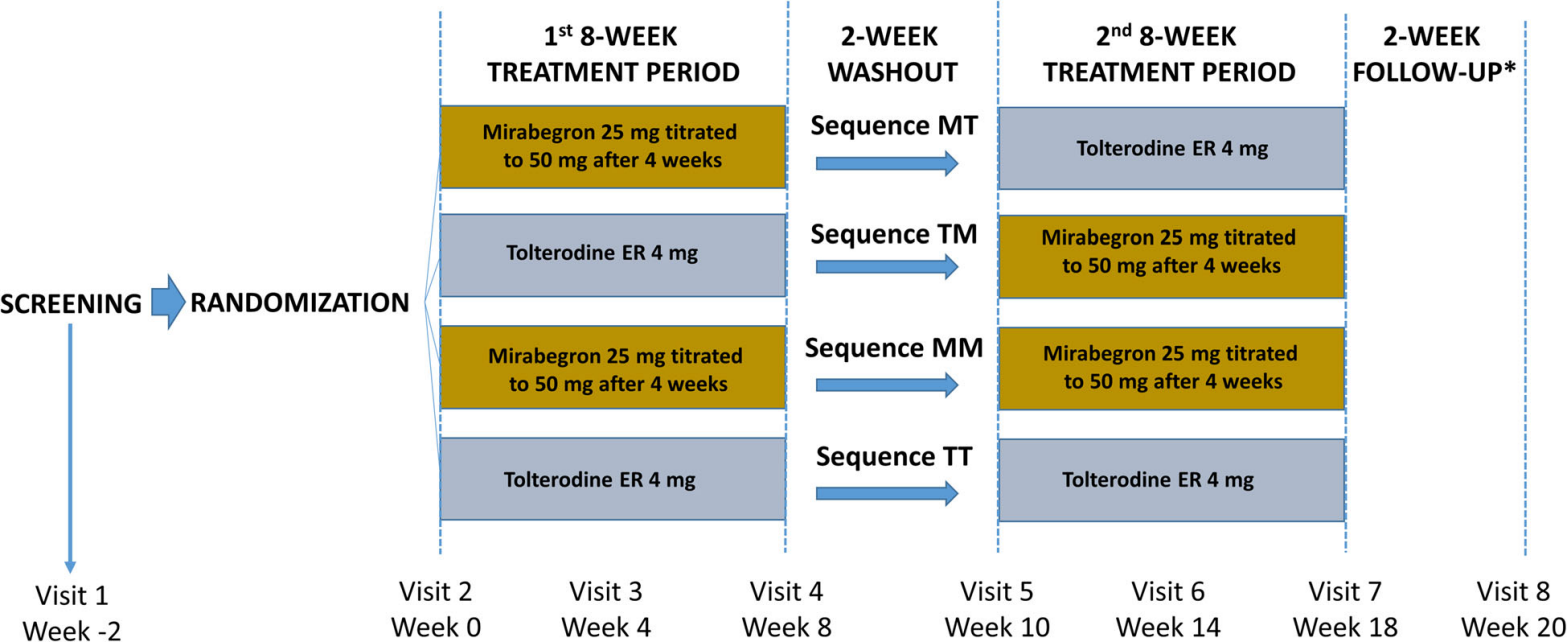
PREFER study design

The study design has been reported previously [18]. In brief, treatment-naïve patients aged ≥18 years with OAB symptoms (urinary frequency and urgency with or without incontinence) for ≥3 months before screening were randomized to 1 of 4 treatment sequences using a 5:5:1:1 ratio (mirabegron [M]/tolterodine 4 mg ER [T]; T/M; M/M; T/T, respectively; Fig. 1).

Based on a 3-day bladder diary, eligible patients had ≥3 episodes of urgency (Patient Perception of Intensity of Urgency Scale [PPIUS] [20] grade 3 or 4) and an average of ≥8 micturitions/24 h at baseline*.* After completing the first 8-week treatment period, patients entered a 2-week washout period followed by a second baseline visit at week 10, which marked the beginning of the second 8-week treatment period. The mirabegron dose was increased from 25 mg to 50 mg after 4 weeks in each treatment period. The tolterodine ER dose was 4 mg throughout each study period.

### Patient-reported outcomes

Patients completed the OAB-S, OAB-q, and the PPBC questionnaires at baseline and at each 4-week follow-up study visit. Some of the OAB-S modules, such as Medication Tolerability, are not completed at baseline but only at follow-up. In both 8-week treatment periods, the OAB-S premedication questionnaire (OAB Control Expectations and Impact of Daily Living with OAB) was completed at baseline only, the OAB-S medication questionnaire (Impact of Daily Living with OAB, OAB Control, OAB Medication Tolerability, Satisfaction with OAB Control, Fulfillment of OAB Medication Expectations, Overall Satisfaction with OAB Medication, Willingness to Continue OAB Medication, and Improvement in Day-to-Day Life with OAB Medication) was completed at each follow-up visit, and the OAB-S medication questionnaire module, Interruption of Day to-Day Life due to OAB, was assessed at baseline and at each follow-up visit. The OAB-S Medication Tolerability scale results were reported previously in the primary analysis of PREFER [18]. Each questionnaire was recorded by the patient on a hand-held electronic device.

A higher score for the OAB-S independent scales (each scale ranges from 0 to 100) and single-item overall assessments (each score ranges from 1 to 5) indicates improved outcome in modules associated with treatment satisfaction [9]. For the OAB Control, Satisfaction with OAB Control and Impact on Daily Living with OAB scales, at least five out of the ten items (50%) in each scale had to be completed to compute a scale score. The OAB-S does not have a published MID score; instead various “responder” definitions have been used [12].

The impact of OAB symptoms on HRQoL and the severity of symptom bother experienced by the patient was assessed by the OAB-q. The OAB-q is a self-reported questionnaire with 33 items, each rated on a 6-point Likert scale, and comprises an 8-item Symptom Bother scale (scores ranged from 0 to 100; higher scores indicate greater symptom bother) and 25 HRQoL items (from the HRQoL subscales Coping, Concern, Sleep and Social Interaction [scores ranged from 0 to 100; higher scores indicate better QoL]) [6]. The HRQoL total score is calculated by summating the individual HRQoL subscale scores [6]. The OAB-q has a well-established MID of 10 points that detects clinically meaningful changes in score. Coyne et al. developed the MID for the OAB-q via distribution and anchor-based analyses. They showed that a greater change score was related to greater patient perceived treatment benefit and satisfaction [21].

The PPBC uses a 6-point Likert scale to rate patient’s impression of their current bladder condition (1 = causes no problems, 6 = causes many severe problems) [8]. Lower scores and negative change indicates improvement in bladder condition.

Changes in mean score over time were analyzed for the OAB-S scales and OAB-S single-item overall assessments, and adjusted change from baseline (see below for details of analysis) to EoT for the OAB-S scales, Impact on Daily Living with OAB, and the OAB-q (total HRQoL, HRQoL subscales, Symptom Bother) and PPBC score.

### Responder analyses

Seven responder analyses associated with PROs were defined and reported: one based on the OAB-S Medication Tolerability scale, and six based on the MIDs for the OAB-q (total HRQoL, HRQoL subscales [Sleep, Social interaction, Coping, Concern], and Symptom Bother). A responder for the OAB-S Medication Tolerability scale was defined as a patient achieving a score ≥ 90 out of 100, and for each OAB-q scale, by the MID, which is defined as an improvement of ≥10 points [21–23]. There is no published MID for the OAB-S, so we selected an OAB-S Medication Tolerability score ≥ 90 to define responder categories in the OAB-S. In order to have a tolerability score of 90, the patient had to select either “I did not have this side effect” or “it did not bother me at all” for all 6 items in the scale.

### Statistical analyses

The sample size calculations for the PREFER study were based on the primary (OAB-S Medication Tolerability score) and key secondary endpoint (patient preference) as reported previously [18]. The Full Analysis Set (FAS) population was used to summarize demographic and baseline characteristics, each OAB-S Scale, OAB-q, PPBC and the seven responder analyses. The FAS included patients who had received ≥1 dose of double-blind study drug and had ≥1 post-baseline value (OAB-S scale, OAB-q, PPBC) in ≥1 double-blind treatment period. Demographic and other baseline characteristics were summarized by descriptive statistics by sequence in period 1 and by overall treatment group (all sequences combined).

The OAB-S scales at the end of each treatment period were analyzed using an ANOVA model with sequence group, study period, period-by-treatment interaction, sex and treatment group as factors and patient-within-sequence as a random term. Because PRO differences other than tolerability were considered exploratory and the study was not powered to detect them, no hypothesis testing was performed. However, the least squares (LS) mean estimate and two-sided 95% CI for the mean difference between mirabegron and tolterodine ER in the Impact on Daily Living with OAB score was derived from the ANOVA model.

Change from baseline in each treatment period at each visit in OAB-q HRQoL subscales and PPBC score were analyzed using the analysis of covariance (ANCOVA) model with sequence group, study period, period-by-treatment interaction, sex and treatment group as factors, baseline as a covariate and subject-within-sequence as a random term. The number and percent of patients who were responders (OAB-q ≥ 10-point improvement or OAB-S Medication Tolerability score ≥ 90) was summarized by treatment group. Outcomes from the responder analyses were not tested for significance.

## Results

### Patient demographics and baseline characteristics

In total, 358 randomized patients received ≥1 dose of double-blind study medication and completed ≥1 post-baseline value (OAB-S scale, OAB-q, PPBC) in at least one treatment period (M/T [*n* = 154], T/M [*n* = 144], M/M [*n* = 30)] or T/T [*n* = 30]). Three-hundred and twenty-nine (91.9%) patients completed the study, and 29 (8.1%) patients discontinued. The discontinuation rate was similar across treatments. Further details on patient disposition and baseline characteristics have been reported previously [18].

Baseline OAB-S, OAB-q scales and PPBC premedication scales were consistent across sequences in period 1 and overall treatment group (Table 1), and were indicative of moderate levels of symptom bother (scores > 50), moderate problems with bladder condition (PPBC > 4), and QoL (total HRQoL ~ 60). The baseline scores for Impact of OAB on Daily Living (~ 50 out of 100) and Interruption of Day-to-Day Life (~ 2 out of 5) suggest significant disruption to daily activities. Baseline values were slightly improved in period 2 vs period 1 suggesting some carryover effect of the previous therapy.

**Table 1:** Patient demographics, OAB characteristics, and baseline PROs

	Period 1				Total	
	M/T (*n* = 154)	T/M (*n* = 144)	M/M (*n* = 30)	T/T (*n* = 30)	Mirabegron^a^ (*n* = 316)	Tolterodine^a^ (*n* = 310)
Women, *n* (%)	116 (75.3)	108 (75.0)	18 (60.0)	20 (66.7)	232 (73.4)	233 (75.2)
Mean (SD) age, years	53.5 (14.8)	52.3 (12.6)	59.0 (13.1)	54.9 (14.8)	53.4 (13.9)	53.2 (13.7)
Age group, *n* (%)						
< 65 years	117 (76.0)	120 (83.3)	20 (66.7)	21 (70.0)	247 (78.2)	243 (78.4)
≥ 65 years	37 (24.0)	24 (16.7)	10 (33.3)	9 (30.0)	69 (21.8)	67 (21.6)
Race, *n* (%)						
White	123 (79.9)	116 (80.6)	24 (80.0)	24 (80.0)	253 (80.1)	248 (80.0)
Black/African American	28 (18.2)	23 (16.0)	4 (13.3)	5 (16.7)	53 (16.8)	53 (17.1)
Asian	2 (1.3)	4 (2.8)	1 (3.3)	1 (3.3)	7 (2.2)	7 (2.3)
American Indian/Alaska native	0	1 (0.7)	0	0	1 (0.3)	1 (0.3)
Other	1 (0.6)	0	1 (3.3)	0	2 (0.6)	1 (0.3)
Ethnicity, *n* (%)						
Hispanic/Latino	33 (21.4)	24 (16.7)	8 (26.7)	6 (20.0)	65 (20.6)	61 (19.7)
Not Hispanic/Latino	121 (78.6)	120 (83.3)	22 (73.3)	24 (80.0)	251 (79.4)	249 (80.3)
Mean (SD) BMI (kg/m^2^)	28.75 (6.65)	29.96 (7.10)	31.25 (8.49)	31.64 (10.98)	29.56 (7.08)	29.70 (7.45)
OAB characteristics	*N = 154*	*N = 144*	*N = 30*	*N = 30*	*N = 341*	*N = 336*
Mean duration of OAB, months (SD)	81.85 (74.34)	75.11 (99.13)	74.06 (84.98)	67.16 (59.96)	76.98 (86.66)	77.57 (84.62)
Type of OAB, *n* (%)						
Urgency incontinence only	65 (42.2)	55 (38.2)	12 (40.0)	14 (46.7)	139 (40.8)	140 (41.7)
Mixed stress/Urgency incontinence with urgency as predominant factor	53 (34.4)	50 (34.7)	10 (33.3)	8 (26.7)	116 (34.0)	112 (33.3)
Frequency/urgency without incontinence	36 (23.4)	39 (27.1)	8 (26.7)	8 (26.7)	86 (25.2)	84 (25.0)
Incontinent patients at baseline of Period 1, *n* (%)						
Wet	117 (76.0)	98 (68.1)	24 (80.0)	22 (73.3)	250 (73.3)	241 (71.7)
Dry	37 (24.0)	46 (31.9)	6 (20.0)	8 (26.7)	91 (26.7)	95 (28.3)
Previous non-drug treatment, *n* (%)						
Yes	6 (3.9)	6 (4.2)	1 (3.3)	3 (10.0)	11 (3.2)	17 (5.1)
No	148 (96.1)	138 (95.8)	29 (96.7)	27 (90.0)	330 (96.8)	319 (94.9)
Baseline PROs	*N = 154*	*N = 144*	*N = 30*	*N = 30*	*N = 337*	*N = 336*
Premedication OAB-S scales, mean (SE)						
Impact on Daily Living with OAB	44.71 (2.37)	48.36 (2.38) [*n* = 143]	40.50 (5.50)	47.33 (5.15)	51.49 (1.67)	53.78 (1.60) [*n* = 335]
Overall Assessment of Interruption of Day-to-Day Life Due to OAB	1.69 (0.07)	1.67 (0.07) [*n* = 143]	1.57 (0.14)	1.77 (0.19)	1.95 (0.06)	2.01 (0.06) [*n* = 335]
OAB-q scales and PPBC, mean (SE)						
Symptom Bother (0–100; higher score indicates greater bother)	61.20 (1.65)	60.83 (1.62)	66.83 (3.79)	59.83 (3.94)	52.67 (1.28)	51.14 (1.28)
Coping (0–100; higher score indicates improvement)	48.04 (2.25)	51.89 (2.23)	47.50 (6.01)	53.58 (4.58)	56.98 (1.59)	59.96 (1.52)
Concern (0–100; higher score indicates improvement)	51.54 (1.97)	51.57 (2.04)	50.86 (4.74)	56.67 (4.30)	59.86 (1.42)	61.60 (1.41)
Sleep (0–100; higher score indicates improvement)	40.99 (2.13)	44.53 (2.23)	37.20 (4.63)	48.53 (5.27)	51.09 (1.59)	52.44 (1.54)
Social Interaction (0–100; higher score indicates improvement)	75.45 (2.05)	77.44 (2.00)	70.00 (4.71)	76.53 (4.41)	78.86 (1.33)	80.54 (1.28)
HRQL total (0–100; higher score indicates better QoL)	53.09 (1.83)	55.44 (1.88)	50.88 (4.69)	58.03 (4.15)	60.99 (1.34)	63.03 (1.31)
PPBC (1–6; higher score indicates deterioration in bladder condition)	4.51 (0.08)	4.54 (0.09) *[n* = 143]	4.47 (0.21)	4.40 (0.15)	4.20 (0.06)	4.16 (0.06) [*n* = 335]

^a^ Patients with the same treatment in two different periods (sequences M/M and T/T) are counted once

### Patient-reported outcomes – OAB-S

**Fig. 2:**
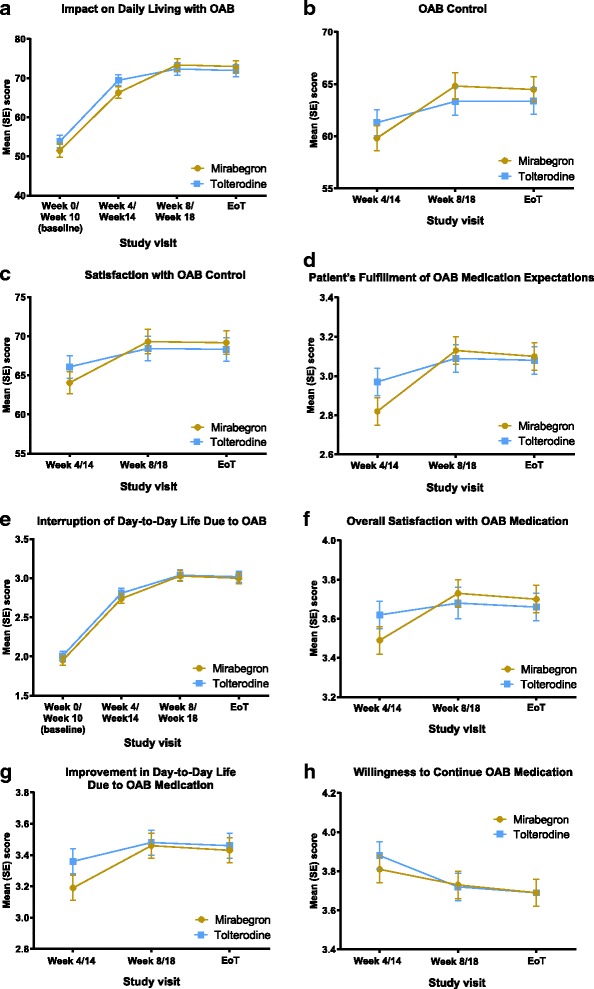
Change in OAB-S scales over time: **a** Impact on Daily Living with OAB; **b** OAB Control; **c** Satisfaction with OAB Control; and change in OAB-S single items: **d** Patient’s Fulfillment of OAB Medication Expectations; **e** Interruption of Day-to-Day Life Due to OAB; **f** Overall Satisfaction with OAB Medication; **g** Improvement in Day-to-Day Life Due to OAB Medication; **h** Willingness to Continue OAB Medication

Improvement over time for the three OAB-S scales (Impact on Daily Living with OAB, OAB Control, and Satisfaction with OAB Control) was similar during treatment with mirabegron and tolterodine ER (Fig. 2a-c). At EoT, the adjusted mean (95% CI) change from baseline was comparable between mirabegron and tolterodine ER, respectively, for the Impact on Daily Living with OAB score (20.41 [16.82, 24.00] vs 19.12 [15.51, 22.73]); an adjusted mean (95% CI) treatment difference of − 1.29 (− 3.79, 1.21). At EoT, the mean (SE) scores were comparable between mirabegron and tolterodine ER, respectively, for OAB Control (64.49 [1.21] vs 63.38 [1.27]) and Satisfaction with OAB Control (69.17 [1.49] vs 68.31 [1.52]).

Similar improvements were observed in four of the five OAB-S single-item overall assessments over time (Fig. 2d-g). At EoT, mean (SE) scores were comparable between mirabegron and tolterodine ER, respectively, for the Patient’s Fulfillment of OAB Medication Expectations (3.10 [0.07] vs 3.08 [0.07]); the Interruption of Day-to-Day Life Due to OAB (3.00 [0.07] vs 3.02 [0.07]); the Overall Satisfaction with OAB Medication Score (3.70 [0.07] vs 3.66 [0.07]); and Improvement in Day-to-Day Life (3.43 [0.08] vs 3.46 [0.08]).

The decrease of Willingness to Continue OAB Medication was similar between mirabegron and tolterodine ER (Fig. 2h): from week 4/14 to EoT, the mean (SE) scores decreased slightly for mirabegron (3.81 [0.07] to 3.69 [0.07]) and tolterodine ER (3.88 [0.07] to 3.69 [0.07]).

### Patient-reported outcomes – OAB-q and PPBC

**Fig. 3:**
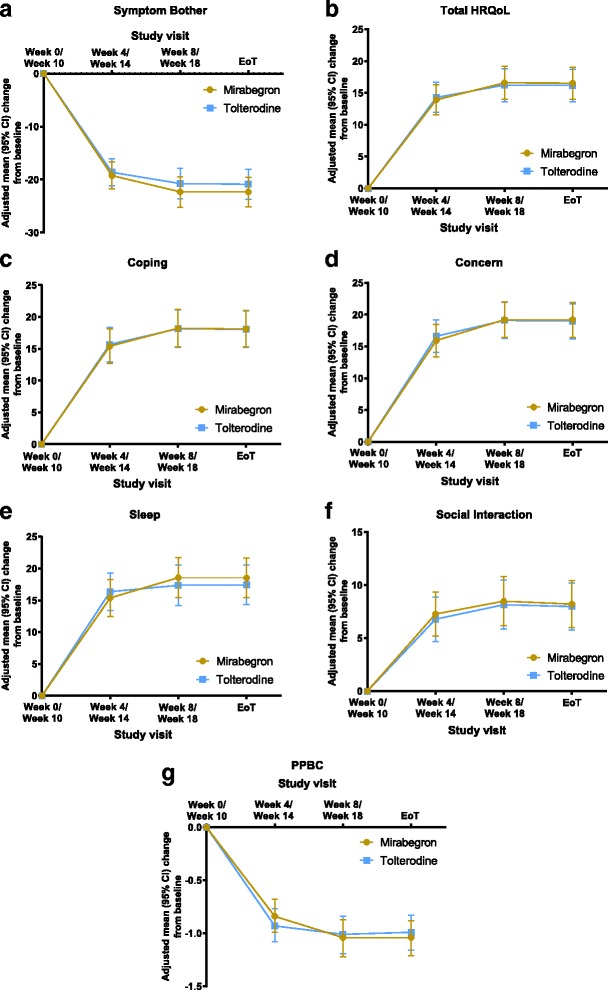
Change from baseline in OAB-q scales and PPBC at each visit. Adjusted change from baseline generated from ANCOVA model with sequence group, study period, period-by-treatment interaction, sex and treatment as factors, baseline as covariate and subject-within-sequence as random term. **a** Symptom Bother; **b** Total HRQoL; **c** Coping; **d** Concern; **e** Sleep; **f** Social interaction; **g** PPBC

Improvements in Symptom Bother score, and total HRQoL and subscales were comparable between mirabegron and tolterodine ER (Fig. 3a-g). At EoT, the mean (95% CI) adjusted change from baseline in the Symptom Bother score was − 22.32 (− 25.13, − 19.52) vs − 20.88 (− 23.69, − 18.06) with mirabegron vs tolterodine ER, respectively; a mean treatment difference (95% CI) vs mirabegron of 1.45 (− 0.64, 3.53). The mean (95% CI) adjusted change from baseline to EoT in the total HRQoL was 16.53 (13.99, 19.06) vs 16.16 (13.61, 18.71) with mirabegron vs tolterodine ER, respectively; a mean treatment difference vs mirabegron of − 0.37 (− 2.13, 1.39). The HRQoL subscales were improved by a similar magnitude (~ 15 points) with the exception of Social Interaction (~ 8 points).

Improvement in PPBC was similar over time. At EoT, the adjusted mean (95% CI) reduction in PPBC score was − 1.04 (− 1.21, − 0.88) for mirabegron vs − 0.99 (− 1.16, − 0.83) for tolterodine ER; an adjusted mean (95% CI) treatment difference vs mirabegron of 0.05 (− 0.11, 0.20). Similar results were obtained at week 8/18 with an adjusted mean (95% CI) treatment difference vs mirabegron of 0.03 (− 0.13, 0.19).

Overall there were no differences in PROs by sequence; however, scores were slightly improved in period 2 vs period 1.

### Responder analyses

**Fig. 4:**
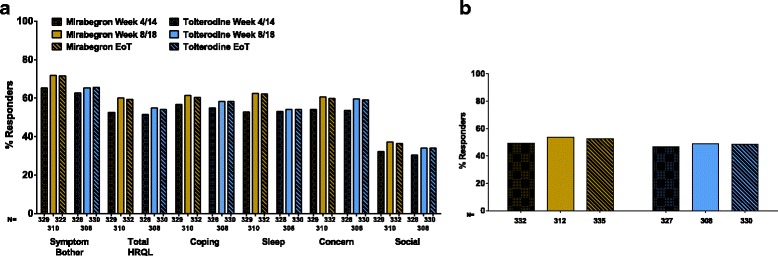
Percentage of responders at EoT. **a** Achieving a ≥ 10-point improvement in OAB-q Symptom Bother score, Total HRQoL, and OAB-q HRQoL subscales; **b** Achieving an OAB-S Medication Tolerability score ≥ 90

For each of the OAB-q subscales, the percentage of responders increased from week 4/14 with similar scores observed at week 8/18 and EoT (Fig. 4a). At each visit, the percentage of responders for each OAB-q subscale was higher for mirabegron than for tolterodine ER. At EoT, the percentage of responders for mirabegron vs tolterodine ER was 71.7% vs 65.5% for Symptom Bother, 60.5% vs 58.2% for Coping, 62.3% vs 54.2% for Sleep, 59.9% vs 59.1% for Concern, 36.4% vs 34.2% for Social Interaction and 59.3% vs 54.2% for total HRQoL (Fig. 4a).

For the OAB-S Medication Tolerability score, the percentage of responders (OAB-S Medication Tolerability score ≥ 90) increased from week 4/14, and was similar between week 8/18 and EoT. At each visit, the percentage of responders was higher for mirabegron than for tolterodine ER; at EoT, the percentage of responders for mirabegron vs tolterodine ER was 52.5% vs 48.5% (Fig. 4b).

## Discussion

The chronic symptoms that characterize OAB have a negative impact on patients’ overall wellbeing, and can manifest as more serious complications such as depression and psychological distress [4]. It is therefore imperative to measure treatment outcomes that reflect how the patient perceives changes in their symptoms and subsequent changes to their daily life. This approach is also more likely to help differentiate OAB treatments, as objective measures of bladder symptoms are usually of a similar magnitude between antimuscarinic agents and mirabegron [13].

In PREFER, which consisted of a treatment-naïve population characterized by moderate levels of symptom bother and disruption to daily activities, the average improvement over time in PROs, as measured by the OAB-S, OAB-q and PPBC questionnaires, were comparable during treatment with mirabegron vs tolterodine ER with the exception of the OAB-S Medication Tolerability score [18]. Although most PROs improved over time, both treatments were similarly associated with a decrease in the OAB-S assessment of Willingness to Continue OAB Medication. The magnitude of change from baseline in most of these scores is either consistent with or greater than changes seen in studies that include non-naïve users [12, 24].

Improvements in the OAB-S questionnaire were evident after 4 weeks of treatment and included positive changes in daily activities, as reflected by scores related to the daily impact of OAB and interruptions to daily routines, and the patient’s perception of treatment benefits according to scores related to satisfaction with OAB control and OAB medication. Treatment expectations may have been higher, and the tolerability threshold lower, in this treatment-naïve population compared with previously treated patients, which may have contributed to the decrease over time in Willingness to Continue OAB Medication that was observed with both study drugs. In the majority of OAB-S scales, the 4-week results with mirabegron were lower than tolterodine ER, but at 8 weeks mirabegron was associated with higher scores than tolterodine ER. This may have been due to a suboptimal duration of treatment for mirabegron at 4 weeks, or that some patients do not respond to the 25 mg dose.

Similar improvements over time with mirabegron and tolterodine ER were evident for each scale of the OAB-q and PPBC. With the exception of the Social Interaction subscale, each OAB-q scale and subscale was markedly improved after 4 weeks of treatment, and by EoT adjusted baseline changes ranged from 15 to 20 signifying clinically meaningful improvements. The PPBC score was reduced by approximately 1, also considered clinically meaningful, after 4 weeks and continued to improve after 8 weeks by a similar magnitude with both treatments. The rapid improvement in the majority of PROs measured at 4 weeks in PREFER reflects temporal improvements in bladder diary parameters as reported in the previous analysis of PREFER, and is consistent with that seen in placebo-controlled phase III trials [18, 25, 26].

Although mean OAB-q scores did not appear to differ between treatments, the responder analyses showed clinically meaningful improvements for the majority of patients (> 50%) in each OAB-q scale except Social Interaction. Although not tested for significance, in each subscale the percentage of responders was higher with mirabegron than with tolterodine ER. The percentage of patients achieving the pre-specified responder definition on the OAB-S Medication Tolerability score (≥ 90) was also higher with mirabegron vs tolterodine ER, supporting the finding of the statistically significant difference in the OAB-S Medication Tolerability score in favor of mirabegron, as reported previously [18]. These responder analyses suggest a greater potential for patients to achieve clinically meaningful improvements in QoL, the perception of their bladder condition, and tolerability with mirabegron.

The magnitude of the improvement in the PROs and the corresponding responder analyses are consistent with subjective endpoints reported in previous phase III studies investigating mirabegron and tolterodine ER [26, 27]. In a recent placebo-controlled phase III trial investigating PROs, mirabegron 50 mg was associated with significant improvements over placebo for two OAB-q subscales (Coping and Concern) which was not observed with tolterodine ER 4 mg [28]. Furthermore, the phase III mirabegron study showed greater improvements in presenteeism and greater reductions in absenteeism and overall work impairment than placebo or tolterodine ER 4 mg [28].

The lack of treatment difference in the average scores on the PROs could be related to the interplay of OAB symptoms and how each individual responds differently according to his or her own priorities, lifestyles and expectations. A real-world evaluation of the OAB-S in older adults starting mirabegron or an antimuscarinic showed similar trends in PRO improvement over time, but without differences between mirabegron and antimuscarinics, despite mirabegron patients being older and having a higher burden of comorbidities and prior treatment [29]. However, failure of the PREFER study to detect these differences over time may have been due to inadequate adjustment for confounders such as comorbidities and age, which may affect PRO outcomes. The randomized crossover higher order design should have accounted for these potential issues and we do believe that any influence of sequence or period on PROs is unlikely given the lack of a period-by-treatment interaction effect on tolerability scores in the previous analysis of PREFER [18]. Despite a 2-week washout period, the slight improvement in PRO scores in period 2 is possibly indicative of some carryover effect, despite far greater than 5 half-lives of drug washout. Moreover, the higher order design, whereby sequences in which patients received the same drug twice were included, allowed within-patient estimates of treatment effect irrespective of carryover effect. Also evident from the previous analysis of PREFER was that tolerability was not the main reason patients gave for their preference of medication at the end of the study, with only 24.7% and 18.7% of patients choosing “tolerated better” during treatment as the reason for their preference with mirabegron and tolterodine ER, respectively [18]. Therefore if patients value improvement in their symptoms over fewer side effects, differences in tolerability are only one piece of the puzzle. The relatively shorter treatment period in this study (8 weeks) compared with most OAB trials (12 weeks) may also have contributed to the lack of treatment differences; for example, patients experiencing side effects such as dry mouth for 8 weeks may not be as bothered as they might after 12 weeks. It is also worth noting that while mirabegron 25 mg demonstrates good efficacy at 4 weeks, maximal efficacy is not reached until 8 weeks and hence the maximal efficacy was reached as the patients were approaching the washout/switch period [30]. A handheld electronic device was used to record PROs. Although this approach has been shown to improve the accuracy and reliability of recording bladder diary parameters versus a paper diary, [31] we have not found validation of the PROs used in this study for the electronic device. The electronic recording of the PROs used in this study may be a potential limitation; however, we would have no reason to believe that the instruments would behave differently in the two time periods in a crossover study.

PREFER is the first late-phase OAB clinical trial to utilize a crossover design and explore PROs related to QoL and satisfaction, including responder analyses to assess clinically relevant improvements. By measuring PROs using three validated questionnaires, PREFER presents a comprehensive analysis of the patient’s experience of mirabegron and tolterodine ER treatment during the 20-week study. Combining the multidimensional concept of treatment satisfaction (OAB-S), aspects of QoL, symptom bother and perceptions of bladder condition ensures that the most important components of the patient’s lifestyle and wellbeing is measured following treatment. Despite previously demonstrating a difference in tolerability in favor of mirabegron, which was accompanied by a reduced rate of anticholinergic side effects, there were no differences in average scores over time between mirabegron and tolterodine ER for other scales of the OAB-S, OAB-q and PPBC. However, the proportion of patients achieving clinically relevant improvements in the overall scores for these questionnaires as measured by the MID or patients who were not bothered by the drug side effect was consistently higher with mirabegron than tolterodine ER.

Sample size calculations for PREFER were based on the primary endpoint (OAB-S Medication Tolerability Score), but not for other outcomes. Specifically because the other PROs were exploratory in nature, hypothesis testing of the other PROs was not done. Although multivariate analysis corrected for sex and baseline values was carried out, it is possible that important covariates such as age and comorbidities that may have had an impact were missed. However, other randomized controlled trials that have used other PROs have not controlled for comorbidities, and have found differences [24].

Phase IV studies of both treatment-naïve and -experienced patients, have demonstrated higher persistence with mirabegron vs antimuscarinics, [16, 32] which may be a consequence of greater tolerability and preference for mirabegron. The relationship between treatment preference, patient behavior and persistence requires further investigation to determine who derives the most benefit from the different treatments.

## Conclusion

Patients with OAB reported improvements in treatment satisfaction, QoL and symptom bother that were of a similar magnitude during treatment with mirabegron and tolterodine ER. A higher percentage of patients achieved clinically relevant improvements in the OAB-S Medication Tolerability score and OAB-q during mirabegron treatment than tolterodine ER. Further studies are recommended to identify factors related to OAB (i.e. symptom severity) and the patient (i.e. age, lifestyle factors) that are predictive of treatment success and longer term persistence and compliance.

## Abbreviations

ANCOVA: Analysis of covariance
CI: Confidence interval
EoT: End of treatment
FAS: Full analysis set
HRQoL: Health-related quality of life
OAB: Overactive bladder
OAB-q: OAB-questionnaire
OAB-S: OAB-satisfaction
PPBC: Patient Perception of Bladder Condition
PREFER: A Prospective, double-blind, Randomized, two-period, crossover, multicenter study to Evaluate the tolerability and patient preference between Myrbetriq® and Detrol® LA in subjects with overactive bladder (OAB)
PRO: Patient-reported outcomes
QoL: Quality of life
SE: Standard error
